# Impact of self-funding on patient experience of oral anticoagulation self-monitoring: a qualitative study

**DOI:** 10.1136/bmjopen-2016-013123

**Published:** 2016-12-23

**Authors:** Alice Tompson, Carl Heneghan, Stephen Sutton, David Fitzmaurice, Alison Ward

**Affiliations:** 1 Nuffield Department of Primary Care Health Sciences, University of Oxford, Oxford, UK; 2 Institute of Public Health, University of Cambridge, Cambridge, UK; 3 Primary Care Clinical Sciences, University of Birmingham, Birmingham, UK

**Keywords:** self-monitoring, self-funding, QUALITATIVE RESEARCH

## Abstract

**Objective:**

To explore the impact self-funding has on patient experience of oral anticoagulation therapy self-monitoring.

**Design:**

Semistructured, qualitative interviews were conducted. Transcripts were analysed thematically using constant comparison.

**Setting:**

England.

**Participants:**

Interviewees were participants of the Cohort Study of Anticoagulation Self-Monitoring (CASM). Cohort members were recruited as they bought a monitor from the major manufacturer in the UK. A purposive sample was invited to be interviewed on completion of the 12-month cohort follow-up.

**Data:**

Patient narratives on their experiences of self-monitoring their oral anticoagulation therapy in non-trial conditions.

**Results:**

26 interviews were completed. Interviewees viewed purchasing the monitoring device as a long-term commitment balancing the limitations of clinic-based monitoring against the cost. They were unable to try out the monitor prior to purchase and therefore had to be confident in their own ability to use it. The variable provision of self-monitoring equipment caused resentment, and interviewees were uncomfortable negotiating with healthcare professionals. High test strip usage while learning how to use the monitor caused anxiety that was exacerbated by worries about their cost. However, self-funding did mean that interviewees felt a sense of ownership and were determined to persevere to overcome problems.

**Conclusions:**

Self-funding has negative implications in terms of equity of access; however, the money invested acts as a barrier to discontinuation. If oral anticoagulation therapy self-monitoring devices and consumables were provided free of charge in routine care, the training and support available in England may need to be reviewed to prevent discontinuation rates rising to those observed in clinical trials.

**Strengths and limitations of this study**

- Qualitative methods allowed an in-depth insight into the patient experience of self-monitoring their oral anticoagulation therapy.
- Patients were self-monitoring in ‘real-life’ and not in trial conditions.
- Interviews were conducted at the end of the 12-month cohort follow-up and the passing of time may have shaped their recollections regarding beginning to self-monitor.
- Given the cost of self-monitoring, interviewees tended to be well educated and from less deprived neighbourhoods and so not representative of all patients that could be offered self-monitoring.

## Background

Patients at increased risk of thromboembolism can be given oral anticoagulation therapy (OAT) using vitamin K antagonists to reduce their risk. Such therapy requires close monitoring and dose adjustment to maximise its benefits. A systematic review and meta-analysis of trial data found that self-monitoring improved the quality of anticoagulation monitoring and reduced the risk of thrombosis by half when compared with clinic-based monitoring.^1^ Furthermore, participants of trials reported improvements in their quality of life.^2–7^

Previously, there have been concerns regarding whether the positive effects found in OAT self-monitoring trials translate into real-life settings. To help address these uncertainties, we conducted a prospective cohort study in the UK (the Cohort Study of Anticoagulation Self-Monitoring (CASM)), which found that individuals can successfully self-monitor their OAT outside of clinical trials, achieving high levels of anticoagulation control and few adverse events,^8^ a finding replicated elsewhere.^9–11^ As part of the CASM study, participant interviews revealed that although self-monitoring made a positive impact on their lives, better health service support would have alleviated some problems.^12^ An unanticipated finding from the interviews was the extent to which self-funding had shaped their experiences.

In England, OAT self-monitoring is a minority activity: most patients attend anticoagulation monitoring clinics either at their local General Practitioner (GP) Surgery or hospital. OAT self-monitoring devices are not provided by the National Health Service (NHS) and so patients wishing to self-monitor obtain them directly for ∼£400. The provision of the consumables required—including the single-use test strips on prescription (ie, heavily subsidised if not free)—and self-monitoring training varies depending under which NHS Clinical Commissioning Group (previously known as a Primary Care Trust) the patient falls. Access to OAT self-monitoring in England is therefore dependent on the patient's ability to pay for it. We could not find any previous in-depth reports in the literature of the effect that funding support has on patient experience of self-monitoring and therefore decided to present this theme separately from the main qualitative findings^12^ to enable a full description.

## Methods

The full methods and results of the CASM study are published elsewhere.^8^ Briefly, 296 people who had decided to self-monitor their OAT were recruited as they purchased a monitor (the Roche Coaguchek S or XS) either as a first or replacement model. Participants were followed up for 12 months with qualitative interviews being conducted at the end of this period in a sample of cohort members approached by telephone. A purposive sampling strategy—based on age, gender, condition requiring OAT, duration of self-monitoring, primary or secondary care setting for anticoagulation management and dose adjustment system—was used to ensure that a range of experiences were studied.^13^ An interview schedule was developed based on the literature and issues that had arisen during the quantitative cohort follow-up.^8^ Analysis ran concordantly with data collection to allow refinement of this schedule. All interviews were conducted by the same non-clinical researcher trained in qualitative research methods (AT) on a one-to-one basis. They were digitally recorded, transcribed and checked to ensure accuracy. The software NVIVO (QSR International) was used to organise the interview transcripts and the coding framework.^14^ Initial coding was undertaken independently by two researchers to ensure that all areas were covered and refined using the constant comparison method.^15^ Both novel and anticipated themes emerging from the transcripts were investigated. Recruitment continued until thematic saturation occurred.

## Results

In total, 26 of the 34 CASM participants approached agreed to be interviewed. Interviews were conducted between November 2011 and November 2012 and lasted between 30 and 75 min in duration. Nineteen interviews were face-to-face (17 at the interviewees' homes, two in their workplace); the remaining seven interviews were conducted on the telephone. The sample had a median age of 54 years (range 32–87 years) and comprised of 10 men (38.5%). The majority had either a professional (11, 42.3%) or university qualification (9, 34.6%) and tended to live in less deprived neighbourhoods (median Index of Multiple Deprivation Score 2010 of 10.4).^12^ Most interviewees (11, 42.3%) required anticoagulation due to previous thrombosis; eight (30.8%) due to having a mechanical heart valve; four (15.4%) because of atrial fibrillation and three (11.5%) had antiphospholipid syndrome.

**Figure 1:**
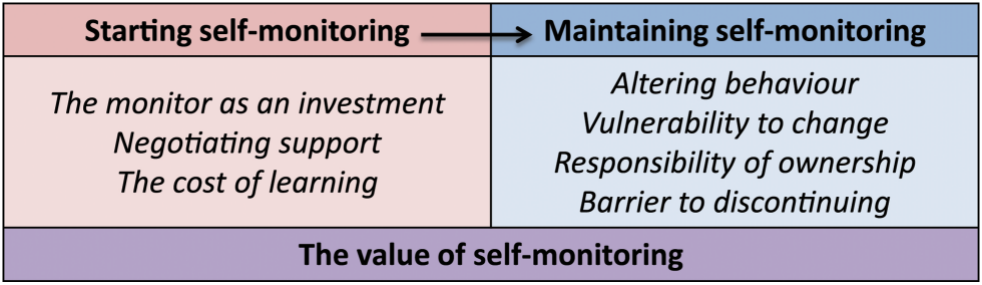
Themes relating to the impact of self-funding on anticoagulation self-monitoring.

Interviewees described how self-funding shaped each stage of their self-monitoring journey from the initial decision to self-monitor and gaining the support required to begin, to motivating them to continue. Throughout this journey, self-funding altered the perceived value of their activities and severity of their condition. Figure 1 describes the themes reported in this paper.

### Starting to self-monitor—the monitor as an investment

Interviewees described balancing the expense of the monitor with the various costs associated with attending the clinic: “Finance is an issue, but then you've got to weigh that up with travelling and petrol and going in the car and waiting, and there's a cost to all that” (ID332, woman, 66 years)*.* Owing to the price of the testing device, beginning self-monitoring was felt to be a long-term commitment: “If I wasn't a lifer [taking warfarin indefinitely] I wouldn't have got one, there would have been no point, it just would have been a waste of money but knowing that I am a lifer then it's worth the money” (ID369, woman, 41 years).

Interviewees researched the device prior to purchase, sometimes using the internet or making contact with other patients already self-monitoring: “I think it's a chance you've got to take…You've got to sort of say, ‘Is it going to work? And how does it work? And is it going to be any good to me?’ And if it is, well, you go ahead and have a go at it…yes, it is expensive. That is the one thing about it. But I think it's worth it” (ID351, woman, 73 years)*.* They were rarely able to gain any practical experience of using the monitor prior to its purchase: “What might have been better is if there had been a system to rent or hire a machine, borrow machine for a period of time just to try it out to see how you get on with it” (ID379, man, 46 years). They, therefore, had to be confident in their own ability to operate it—for example, being dexterous enough to prick their own finger or read the monitor display—in order to invest: “I might have spent £300 and I couldn't use it but that was my decision and that puts people off, the price” (ID307, woman, 59 years).

The decision to spend the money was made in the context of their lives: “I thought well I haven't got that straightaway, it's gonna take me time to earn that, I mean now we've got six children” (ID263, woman, 54 years). For some, the cost required periods of saving or sacrifice: “If somebody's had a stroke and they're out of work and they've not got any financial back-up system, £300 or £400…it seems too much money” (ID307, woman, 59 years). The interest free repayment scheme or special discounts offered by the monitor manufacturer were welcomed: “I'm paying for the actual thing, you know, I had it over the year. Thank goodness, it was zero interest” (ID340, woman, 78 years).

### Starting self-monitoring—negotiating support

Interviewees were largely resigned to funding their own monitor. However, the uncertainty surrounding the supply of consumables on prescription caused concern when arranging to start self-monitoring: “They did say before you get one [monitoring device] make sure your GP will prescribe the test strips and the lancets because a lot of them don't, which is what I did first, I did speak to the doctors first and then they had a meeting and they decided they would so then I went ahead and ordered one” (ID369, woman, 41 years). Having rationalised the cost of the testing device by intending to self-monitor for a continued period, the cost of testing consumables required would not be insignificant: “If I'd of have had to buy the test strips as well I would have thought twice about it. The fact that I could get those on prescription and the lancets as well means the running costs, apart from batteries, are negligible to me” (ID379, man, 46 years).

Those in contact with patient support groups entered into negotiations for testing consumables on prescription conscious that their healthcare professional may decline their request*.* The decision of the local commissioning body was often delivered by interviewees' GPs: “They don't prescribe the strips in this area because it's a postcode lottery…but my GP told me that at the beginning” (ID339, woman, 49 years). The negotiations took place amid ongoing healthcare sometimes causing anxiety: “I was so upset the way that I've been spoken to, I'm too frightened they'll mark my card and get rid of me from their surgery” (ID281, woman, 42 years)*.* Several interviewees reported changing healthcare professional or conversely delayed moving in order to be able to access support to self-monitor: “I don't think any patient should be involved in politics really and I feel that I've very much been…in the difficult position of… I don't like facing conflict face-to-face, I mean very few of us do” (ID350, man, 32 years).

When negotiating the supply of testing consumables, interviewees described their healthcare professionals as apprehensive of excessive testing: “The only thing that was worrying her [the GP] was whether I would get a bit obsessive about doing the finger prick constantly and therefore asking her to pay for rather expensive…test strips” (ID293, woman, 73 years). Owing to these concerns, some healthcare professionals rationed the supply: “I'm allowed to have twenty-four a year from them [the GP surgery]” (ID369, woman, 41 years).

### Starting self-monitoring—the cost of learning

All interviewees described an initial period of mastering how to apply blood to the test strip correctly. During this time, a number of strips were wasted and usage was higher than expected: “I got cross, I got upset. Not just because of the price of the strip, but because I couldn't do it and I wanted to know why” (ID351, woman, 73 years). Those that paid for their own strips experienced the consequential costs directly: “I floundered at first…I kept thinking, ‘Oh not another strip, I've got to use another strip, they're so expensive’’’ (ID340, woman, 78 years). Those that received their test strips on NHS prescription were worried that this initial high consumption would lead to this privilege being withdrawn: “I remember, every time I wasted a strip, I felt very angry because I was in a context of feeling that the supply of strips was very limited and the doctor wasn't happy to give them to me” (ID350, man, 32 years)*.* Once the initial learning period had passed, interviewees were able to use the monitor more reliably and strip usage decreased.

### Maintaining self-monitoring—altering behaviour

Among our sample, there was limited evidence that having to pay for the test strips restricted the amount they were able to test: “It will get to the point where you think, ‘I'm not going to do it because I can't test when I should be because I can't afford to do it’. So that's what worries me long term” (ID281, woman, 42 years). More typical—among this relatively affluent group—was that by self-funding interviewees felt able to disregard healthcare professional advice and test more frequently in order to keep a closer eye on their anticoagulation status: “I'm paying for the strips, so [laughs] they wouldn't know whether I was doing it or not!” (ID351, woman, 73 years).

### Maintaining self-monitoring—vulnerability to change

Among those that had successfully negotiated to receive test strips, there was an apprehension that this decision was temporary: “I was actually managing to get the strips through negotiation, but a little thing like when I went to fill in the repeat prescription forms, they were never listed there for me to tick, I had to actually write on the form ‘and I need some test strips’…it felt each time that I was almost being given them as goodwill and it was going to end soon” (ID350, man, 32 years)*.* A handful of interviewees reported that the decision to supply test strips on prescription was subsequently reneged by the Clinical Commissioning Group citing financial pressures. Interviewees were not consulted about these decisions and felt passive in this process: “They've never discussed the matter with me before writing a letter saying, ‘There's the guillotine’” (ID211, man, 78 years).

### Maintaining self-monitoring—responsibility of ownership

With patients owning their testing device and their anticoagulation clinic using the results, taking responsibility for ensuring accuracy of the monitor was a grey area. Following a calibration check in which her monitor was compared to a venous test at the clinic, one interviewee described how neither the manufacturer nor the clinic were willing to help her resolve the large difference between results: “He [the manufacturer representative] would not admit that there could be anything wrong with the machine, that it had to be at the hospital's end” (ID263, woman, 54 years)*.* Self-monitoring contracts between clinics and patients to set out roles and responsibilities were valued. However, not all interviewees had these or they did not include responsibility for resolving accuracy issues. Interviewees also reported uncertainty about the lifespan of their monitor: “Does it get inaccurate after a period of time?…How long does it last?”(ID352, man, 55 years); “I checked the warranty of it because this is another factor, fortunately it has a two year warranty which if it packs up what do you then do?” (ID239, man, 68 years).

### Maintaining self-monitoring—a barrier to discontinuation

While self-funding had implications in terms of equity of access, it meant that interviewees were determined to persevere. For example, when encountering problems using the monitor initially, interviewees felt compelled to continue: “Let's just keep going…and we'd also spent whatever it was” (ID342, man, 48 years). For those who had had their supply of test strips on prescription discontinued, by this point they had purchased their testing device and often established self-monitoring routines. Given the amount of time, effort and money already invested they felt they had little choice but to incur the additional costs and to continue to self-monitor. “I'd stick with the machine now…I've paid for the machine” (ID387, man, 47 years).

### The value of self-monitoring

The variation in the support OAT self-monitoring patients received caused resentment: “I know you can't put a price on your health but when it is available on prescription and everybody else gets it that's when it's unfair” (ID281, woman, 42 years). The situation was often contrasted against that for diabetes: “The test strips aren't exactly cheap but then diabetics get everything, they get all their stuff for free don't they? So why can't we have a helping hand then?” (ID369, woman, 41 years). Some felt that diabetes was therefore viewed as more serious: “They don't think that having a blood clotting disorder is life threatening, how can that not be? Diabetes is life threatening but I'm sorry, the blood that I've got is also, you know, life threatening” (ID357, woman, 55 years). Others highlighted the similarities on the impact to their lives: “It's [Warfarin's] like being on insulin, that's a good comparison really because insulin you have to take every day, Warfarin for life you take every day. They're both quite dangerous materials” (ID307, woman, 59 years).

Interviewees felt that the benefits to the NHS of their self-monitoring activities were unacknowledged and unappreciated: “You're taking over the hospital's role and you're funding it yourself, they're saving money on you but you're paying for it” (ID281, woman, 42 years). This included taking responsibility for monitoring their long-term health condition and avoiding adverse events: “Get a monitoring kit and you've got to control that and do your bit towards the NHS really by not going to casualty phlebotomy clinic you're saving a lot of money and also your own time” (ID307, woman, 59 years). By declining to fund it, it was felt that the NHS did not value the effort they expended self-monitoring.

## Discussion

### Summary

The CASM study sought to explore the factors that predict success and failure in self-monitoring of OAT outside of clinical trial conditions. We anticipated that psychological and social factors would be important. While they played a role, we found that in the ‘real world’ the variable healthcare support available was key in shaping patient experience, an important aspect of which was the provision of the equipment and consumables required.^8^ ^12^

Unlike clinical trial participants, interviewees purchased their own monitor. The price caused careful reflection on their ability to self-monitor and possibly filtered out those less determined or lacking self-efficacy. Interviewees felt a sense of ownership and the money invested acted as a barrier to discontinuation especially during the problematic initial stages. The cost of the test strips caused anxiety, especially when usage was higher while the correct testing technique was learnt.

### Strengths and limitations

The qualitative component of the CASM study is the first to investigate in depth the patient experience of OAT self-monitoring in a non-trial, UK setting. Participants were interviewed about their experiences at the end of the 12-month cohort follow-up and it is possible that the passing of time altered their recollections of starting to self-monitor. Unsurprisingly, given the cost of the monitor, interviewees typically lived in less deprived neighbourhoods and are not representative of all anticoagulation patients that could self-monitor. Therefore, while it has not been possible to study the full breadth of the impact patient self-financing could have on patient experience of self-monitoring, the study provides an insight into this previously unreported area.

Since this study was conducted, the use of novel oral anticoagulants in clinical practice has increased. This class of drugs offers an alternative for patients not wishing to attend regular monitoring appointments or to self-monitor. However, they are not suitable for patients with severe kidney disease, impaired liver function or a prosthetic heart valve.^16^ Furthermore, some patients may be reluctant to switch due to the lack of commercially available antidotes for some novel oral anticoagulants^17^ meaning that self-monitoring will continue to play a role in anticoagulation management.

### Comparison with existing literature

Among our relatively affluent sample, there was very limited evidence of self-testing frequency being altered by concerns about cost. This is in contrast with several studies of patients with diabetes conducted in health systems where self-monitoring consumables were not supplied free of change. Non-adherent self-monitoring was found in patients with higher out-of-pocket costs for glucometer strips in California^18^ while the price of test strips was identified as a major barrier to self-monitoring in Trinidad and Tobago^19^ and Malaysia.^20^ A qualitative study conducted in Canada found evidence of patients rationing test strips and reusing lancets in order to reduce costs.^21^

It is recognised that self-management can be hard work for patients, even with well-developed support systems in place.^22^ Along with the more typical challenges of learning a new skill and developing a routine,^12^ our interviewees expended additional effort and energy trying to gain healthcare professional support and the equipment required to be able to self-monitor.

While self-funding may deter all but the most committed patients from attempting to self-monitor their OAT, it also means that access to the clinical and broader benefits is limited to those that can afford to do so. Restricting access to those patients able to pay rather than those with the most to gain from self-monitoring is inequitable and sits uncomfortably against the founding principles of the NHS, a system intended to be free at the point of delivery.

### Implications for practice and research

Anticoagulation clinics in England could offer patients an opportunity to try a monitor prior to purchase to make their subsequent investment less of a gamble. In terms of the consumables required, focusing on the cost of testing strips places an additional burden on patients learning how to use the monitor. Patients need reassurance that this learning phase is normal, and allowance should be made if calculating the initial ‘ration’ of test strips. Hands-on training could be more widely offered in England so that the correct technique can be mastered with minimal stress. The use of privately funded monitors within the context of ongoing NHS care is a relatively new phenomenon and creates some difficulties.^23^ Further guidance regarding who is responsible for ensuring the accuracy of the testing devices and resolving any technical problems is required. This information could be added to the self-monitoring contracts patients are required to sign by some anticoagulation clinics.

If testing devices were to be provided by the NHS in England, the variable availability of training schemes and the robustness of support systems should be reviewed. While the current arrangements appear adequate for the highly motivated patients who are able to overcome the existing barriers in place to begin self-monitoring, this may not be the case if access is made easier. Improved support would help prevent discontinuation rates rising to those observed in clinical trials of OAT self-monitoring.^24^ Future studies could investigate healthcare systems in which anticoagulation self-monitoring equipment is provided for patients—such as Germany—to help inform the design of this support.

The impact of the provision of self-monitoring equipment to trial participants in other conditions such as hypertension could also be explored to improve the generalisability of research findings to clinical practice.
